# Natural Compounds Targeting Oncogenic Signaling Networks in Glioblastoma: Molecular Mechanisms, Translational Advances, and Clinical Perspectives

**DOI:** 10.1002/brb3.71689

**Published:** 2026-08-26

**Authors:** Mehrukh Zehravi, Md Abul Hassan, Md. Al Amin, Sherouk Hussein Sweilam, Jeetendra Kumar Gupta, Ramenani Hari Babu, Pericharla Venkata Narasimha Raju, Krishna Vamsi Kandimalla, Phanindra Erukulla, Patibandla Jahnavi, Rajeshwar Vodeti, Abdul Ajeed Mohathasim Billah, Safia Obaidur Rab, Sharuk L. Khan

**Affiliations:** ^1^ Department of Clinical Pharmacy College of Dentistry & Pharmacy, Buraydah Private Colleges Buraydah Saudi Arabia; ^2^ National Research Center For Protozoan Diseases Obihiro University of Agriculture and Veterinary Medicine Obihiro Japan; ^3^ Department of Pharmacy State University of Bangladesh Dhaka Bangladesh; ^4^ Department of Pharmacy, Faculty of Health and Life Sciences Daffodil International University Dhaka Bangladesh; ^5^ Department of Pharmacognosy College of Pharmacy Prince Sattam Bin Abdulaziz University Al‐Kharj Saudi Arabia; ^6^ Department of Pharmacognosy, Faculty of Pharmacy Egyptian Russian University Cairo Egypt; ^7^ Institute of Pharmaceutical Research GLA University Mathura Uttar Pradesh India; ^8^ Amity Institute of Pharmacy Amity University Gwalior Madhya Pradesh India; ^9^ Department of Regulatory Affairs Hikma Pharmaceuticals USA Inc. Cherry Hill New Jersey USA; ^10^ Department of Regulatory Affairs InvaGen Pharmaceuticals Central Islip New York USA; ^11^ Department of Regulatory Affairs Ricon Pharma LLC Denville New Jersey USA; ^12^ Department of Pharmaceutics KVSR Siddhartha College of Pharmaceutical Sciences Vijayawada Andhra Pradesh India; ^13^ Department of Pharmaceutics, School of Pharmacy Anurag University Hyderabad Telangana India; ^14^ Department of Pharmacy Practice, Sri Ramachandra Faculty of Pharmacy Sri Ramachandra Institute of Higher Education and Research Chennai Tamil Nadu India; ^15^ Department of Clinical Laboratory Sciences College of Applied Medical Science King Khalid University Abha Saudi Arabia; ^16^ Department of Pharmaceutical Chemistry N.B.S Institute of Pharmacy Latur Maharashtra India

**Keywords:** glioblastoma, molecular insights, natural compounds, signaling pathway

## Abstract

**Purpose:**

Glioblastoma (GBM), the most aggressive and common primary brain tumor in adults, has a poor prognosis, rapid growth, and resistance to treatment. Abnormal activation of signaling pathways like PI3K/AKT/mTOR, MAPK/ERK, JAK/STAT, Wnt/β‐catenin, NF‐κB, and Notch facilitates uncontrolled proliferation, angiogenesis, invasion, and immune evasion. Conventional treatments are insufficient in modifying complex networks, necessitating the urgent need for novel multitargeted treatments. Natural compounds are increasingly being considered as potential treatments for GBM due to their ability to control multiple oncogenic pathways simultaneously and their lower toxicity compared to synthetic medicines. This review provides an integrated and translational perspective on GBM, differing from past reviews that focused on the anticancer effects of individual phytochemicals or specific signaling pathways. We discussed the relationship between natural compounds and key oncogenic signaling networks, focusing on GBM pathogenesis, blood–brain barrier penetration, nanotechnology‐assisted delivery, and strategies combining standard therapies. Furthermore, this review critically distinguished in vitro, in vivo, and clinical evidence, focusing on bioavailability challenges, ongoing clinical trials, and future opportunities in precision oncology.

**Method:**

To ensure the inclusion of the most appropriate articles in this review, an in‐depth search was carried out on prominent medical, biological, and chemical databases, including Scopus, PubMed, and Web of Science. The search strategy employed combinations of Medical Subject Headings and Boolean operators, focusing on terms related to glioblastoma, natural compounds, and various molecular mechanisms involved in drug delivery, clinical trials, and diagnosis.

**Finding:**

These natural compounds demonstrated potential in preclinical GBM models for inducing apoptosis, suppressing angiogenesis, modulating oxidative stress, and enhancing chemosensitivity. Additionally, some natural compounds can cross the blood–brain barrier, thereby increasing their translational significance. The use of molecular diagnostics, including biomarkers, genetic profiling, and advanced imaging, has significantly improved early identification and patient stratification. Specific compounds with strong translational potential, such as curcumin, resveratrol, EGCG, quercetin, berberine, and luteolin, are highlighted along with their main molecular targets and therapeutic effects. The integration of natural compounds into precision‐based therapy approaches has been made possible.

**Conclusion:**

Future research aims to combine molecular diagnostics with phytochemical therapies, validate clinical trial outcomes, and enhance bioavailability with nanotechnology delivery systems. The combination of natural compounds shows potential for creating safe, effective, and pathway‐specific treatments for GBM.

## Graphical abstract

Glioblastoma (GBM) is a highly aggressive brain tumor with a poor prognosis, driven by abnormal signaling pathways that promote growth and resistance to treatment. Conventional therapies are inadequate, highlighting the need for novel multitargeted approaches. Natural compounds are emerging as promising treatments due to their ability to target multiple oncogenic pathways with lower toxicity. This review explores the relationship between these compounds and GBM pathogenesis, delivery methods, and integration with standard therapies.

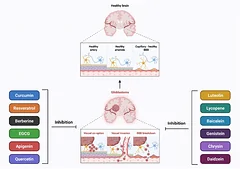

## 1 Introduction

Glioblastoma (GBM) is the most prevalent primary malignant brain tumor, with an incidence rate of 3.23 per 100,000 population (Pellerino et al. 2022). GBM is a cancerous growth that develops from astrocytes and affects the brain or spinal cord. Primary brain tumors are the most prevalent type, with occurrence rates of 3.19 cases per 100,000 patients in the United States and 2.05 cases per 100,000 in the United Kingdom (Tamimi and Juweid 2017). The most prevalent malignant tumor of the central nervous system (CNS) in adults is GBM. In the United States, it is responsible for about 14.5% of all CNS neoplasms, 48.6% of all primary malignant CNS tumors, and 57.7% of gliomas. After adjusting for the standard population, the annual incidence of GBM in the United States is 3.23 per 100,000 people (Ostrom et al. 2020). Brain tumors are a major cause of morbidity and death, causing significant disability and financial burden on families and healthcare systems. The incidence is estimated at 25.48 per 100,000 (de Robles et al. 2015). The incidence of GBM is 3.19 per 100,000 persons annually in the United States (Dolecek et al. 2012), whereas it is 0.85 per 100,000 persons annually in Taiwan (Chien et al. 2016). The average annual incidence rate for primary brain tumors is about 24.25 per 100,000, with malignant tumors at 6.94 and nonmalignant tumors at 17.88 (Ostrom et al. 2022). The Consortium to Inform Molecular and Practical Approaches to CNS Tumor Taxonomy and the fifth edition of the WHO Classification of Tumors of the CNS have updated GBM nomenclature to align solely with wildtype IDH status, moving away from its previous classification into IDH‐wildtype and IDH‐mutant groups (Chen et al. 2022). GBM survival estimates are significantly low, even when full surgical excision, radiation therapy, and chemotherapy are used. Newly diagnosed patients with GBM have a mean survival duration of 12–15 months, a 2‐year survival rate of 13%–26%, and a 5‐year survival rate of less than 5% (Hegi et al. 2005). The poor prognosis of patients with highly malignant brain tumors is largely due to their invasive and diffuse nature (Furnari et al. 2007). Despite advancements in GBM treatment, numerous challenges remain, especially in traditional therapeutic methods. GBM's widespread invasion and poorly defined tumor boundaries make surgical tumor removal less effective despite increasing survival rates (Lima et al. 2012; Ringel et al. 2015). GBM's difficulties primarily stem from the molecular and genetic signaling pathways that lead to tumor development. The genes for cyclin‐dependent kinase inhibitors (e.g., CDK2NA) are deleted, the O‐6‐methylguanine‐DNA methyltransferase (MGMT) gene is silenced, and the epidermal growth factor receptor (EGFR) and cyclin‐dependent kinase (e.g., CDK4) genes are amplified in GBM (Balça‐Silva et al. 2019). Clinical trials can be beneficial for different chemotherapy regimens that usually require high medication dosages. Chemotherapy's effectiveness is often limited due to various factors, similar to those of surgery and radiation. Chemotherapeutic treatments typically only partially penetrate the brain due to the difficulty of medications passing across the blood–brain barrier (BBB). The diversity of brain tumor cells means that even if a medication effectively kills some targeted cells (Weller et al. 2013). Chemotherapy faces limitations due to toxicity and the risk of cancer cells becoming resistant to anticancer agents, which can lead to recurrence and potentially fatal disease progression. The combination of these factors significantly influences the frequent restrictions and short‐term advantages of chemotherapy treatments (Boiardi et al. 1991; B. Jeremic et al. 1992). Terpenoids and polyphenols affect tumor development and chemoresistance through the PI3K/Akt/mTOR pathway (Afshari et al. 2025). Natural compounds that control microRNAs (miRNAs) have revolutionized the treatment of brain cancer. Resveratrol and curcumin have demonstrated anticancer properties by modulating miRNA expression associated with treatment resistance and tumor growth. These natural compounds target these miRNAs, increasing treatment efficacy and reducing side effects (Doghish et al. 2025). Curcumin, quercetin, berberine, and resveratrol are phytochemicals with potential anticancer properties, including the induction of apoptosis and inhibition of cell migration and proliferation. Research on pharmacokinetics and cellular effects in GBM and medulloblastoma highlights their potential synergy with conventional treatments. Additionally, emerging studies on nanotechnology show promise for enhanced delivery of therapeutic compounds in these cancers (Ozbek et al. 2026). Additionally, natural compounds are potential chemotherapeutic agents, a crucial component of the lifestyle medicine approach to chronic diseases. These compounds exhibit anti‐GBM actions by upregulating apoptosis and autophagy, disrupting the cell cycle, preventing proliferation, neuroinflammation, chemoresistance, angiogenesis, and metastasis. Natural compounds' effectiveness in GBM is limited by BBB permeability and bioavailability, prompting the development of different chemical formulations to improve their pharmacological properties (Zhai et al. 2021). Cancer molecular signaling events, including cell cycle arrest, DNA topoisomerase suppression, proteasome inhibition, survival/proliferation control, topoisomerase downregulation, fatty acid suppression, and p53 accumulation, play a positive role (Snyder and Gillies 2003). Natural compounds may serve as remedies for GBM by reducing oxidative stress (OS), preventing proliferation, and enhancing antitumor treatments. Focusing on abnormal cellular pathways could lead to novel treatments, improving patients' quality of life and prolonging survival (Behl et al. 2021). The majority of published evaluations of natural compounds for GBM primarily focus on specific phytochemicals or isolated molecular processes, often neglecting their translational significance. GBM pathogenesis, molecular diagnostics, interconnected oncogenic signaling networks, BBB permeability, nanotechnology‐based delivery systems, combination strategies with current standard therapies, and available clinical evidence are all uniquely combined in this review. Furthermore, this review provides comparative insights for the clinical development of natural chemicals aimed at precision GBM therapy, differentiates between preclinical and clinical data, and discusses translational constraints along with future therapeutic prospects.

## 2 Methodology

A literature search identified studies on the therapeutic potential of natural compounds in GBM. Electronic databases, including PubMed, Scopus, Web of Science, and Google Scholar, were systematically searched for articles published up to 2026. The search strategy used combinations of Medical Subject Headings terms and Boolean operators, including “glioblastoma” OR “glioblastoma multiforme” OR “GBM” AND “natural compounds” OR “phytochemicals” OR “plant‐derived compounds” OR “flavonoids” OR “polyphenols” AND “signaling pathways” OR “molecular mechanisms” OR “blood–brain barrier” OR “drug delivery” OR “diagnosis.” Original research articles, systematic reviews, meta‐analyses, and clinically relevant review articles from peer‐reviewed journals were included in the study. Eligible studies focused on molecular mechanisms, modulation of oncogenic signaling pathways, BBB permeability, nanotechnology‐based delivery systems, combination therapies, molecular diagnostics, preclinical findings (in vitro and in vivo), and clinical evidence concerning natural compounds in GBM. Articles not in English, including conference abstracts, editorials, letters, book chapters, dissertations, non‐peer‐reviewed publications, duplicates, and studies irrelevant to the review's scope were excluded. Studies were screened for eligibility by first removing duplicates, then evaluating titles and abstracts independently, followed by a full‐text review. Relevant publications were found by manually screening reference lists of selected articles.

## 3 Pathogenesis of GBM

**FIGURE 1:**
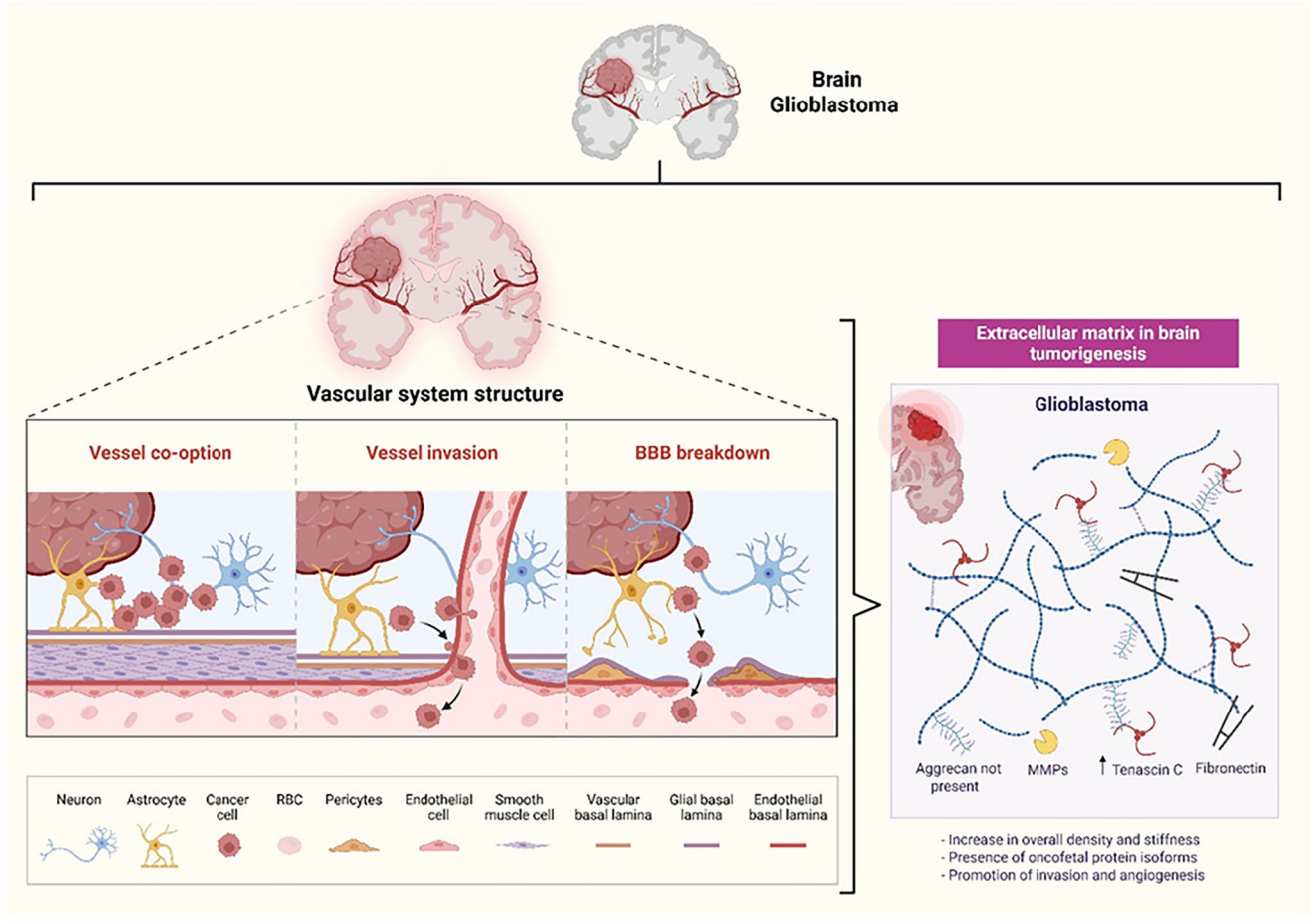
Illustration of the vascular system structure of the brain glioblastoma and ECM in brain tumorigenesis.

One enzyme that is essential to the citric acid cycle is isocitrate dehydrogenase (IDH). IDH1, IDH2, and IDH3 are the three isoforms of IDH. Peroxisomes and the cytoplasm contain IDH1, whereas the mitochondrial matrix contains IDH2 and IDH3 (Fedøy et al. 2007). A study found that nearly all cases of secondary GBM had IDH mutations. IDH mutations are uncommon in initial GBM but prevalent in grade II and III gliomas and subsequent GBM (Parsons et al. 2008). Regular enzymatic function is both lost and gained in the IDH mutation (Cohen et al. 2013). It prevents isocitrate from being converted to α‐KG by decreasing its binding affinity for isocitrate. Cancerogenesis is caused by the aberrant build‐up of the oncometabolite 2‐HG (Turkalp et al. 2014). Mutant IDH inhibitors, like AGI‐5198, help separate cancer cells from proliferating ones, with AG‐120 being the first clinically proven mIDH1 inhibitor (Popovici‐Muller et al. 2018). AG‐5198, a specific R132H‐IDH1 inhibitor, significantly reduced mIDH1's ability to generate 2‐HG and stimulate the expression of gliogenesis‐related genes (Rohle et al. 2013). Furthermore, determining the malignant phenotype of gliomas is linked to their progression. Notch‐2 and Notch‐3, both promoting glioma cell growth, have been identified as predictive markers for gliomas. A correlation between tumor aggressiveness and Notch‐4 was discovered (Yan et al. 2019). Research indicates that stem‐like glioma cells, which contain Notch family genes, can be targeted effectively using the Notch pathway (Fan et al. 2010). Retinoic acid can impact stem‐like cells from GBM, leading to cell growth inhibition, differentiation, and loss of the stem cell pool (Ying et al. 2011). Malignant gliomas have a poor prognosis due to their prevalence and chemoresistance. The kynurenine pathway catabolism of L‐tryptophan produces essential metabolites like QUIN, PIC, and 3‐HAA, which can induce neurotoxicity and suppress antitumor immunity in human tumors, suggesting targeting the KP could prevent these effects (Adams et al. 2012). Malignant brain tumors are largely caused by genetic and epigenetic alterations. DNA methylation state, particularly 5‐hydroxymethylcytosine (5hmC), is crucial for gene expression. Glioma cells use metabolic alterations to increase survival and inhibit immune responses, but can also destroy healthy tissues. Metabolic alterations could lead to new strategies to harm tumor cells and redirect host antitumor reactions, ultimately eliminating deadly tumors (Sowers et al. 2014). Recent genomics has linked these tumors to specific molecular networks, suggesting targeted medicines might be more effective. These tumors are heterogeneous at the cellular level, with stem‐like characteristics, making them resistant to treatment. Treatment issues in these tumors arise from genetic and cellular heterogeneity, including the BBB's resistance to drug delivery and innate and learned factors (Huse and Holland 2010). The mammalian brain consists of billions of neurons and glial cells, with neurodegeneration typically arising from molecular disruptions. Neoplastic transformation is driven by cellular and genetic variables in glial cells and neurons. Microenvironmental roles and regulatory networks rule cellular transcriptome changes during disease development. miRNAs rule genes controlling neural stemness, differentiation, apoptosis, projection fates, and migration. The brain expresses more miRNAs than any other mammalian organ, with expression declining during oncogenesis and increasing during early stages of neurodegeneration. These complexities are crucial for therapeutic restoration in neurodegeneration and determining proapoptotic and pro‐survival signaling in brain miRNA networks (Godlewski et al. 2019). A multi‐omics study reveals SNP rs615552_AL359922.1 controls gene expression and is linked to CDKN2A, CDKN2B, and CDKN2B‐AS1. It also identifies TERT as a gene with shared susceptibility to brain cancer, potentially leading to therapeutic targets and diagnostic instruments (Ye et al. 2025). A new trypsin inhibitor (P25TI) was isolated from human GBM cells using PCR screening. The resulting cDNA encoded 258 amino acids, similar to proteins in mammalian testes, insect venom allergens, and plant disease. P25TI mRNA is broadly expressed in human neuroblastoma and GBM cell lines, as well as in the brain, placenta, and lymphocytes (Yamakawa et al. 1998). The ERK pathway is crucial for promoting tumor characteristics and is linked to EGF receptor activation. The JNK pathway, triggered by cytokines and stressors, is more uniformly activated. However, its role in primary tumors is uncertain. The study investigated the function of JNK in primary human brain tumors and tumor‐derived cell lines to understand its role in carcinogenesis. Results showed that primary brain tumors often exhibit increased JNK activity, contributing to transformation‐related characteristics. The EGF receptor is often overexpressed in primary human brain tumors (Antonyak et al. 2002). MiRNA dysregulation is a major factor in the oncogenesis of cancers, including gliomas. MiRNAs serve as effective biomarkers for gliomas due to their ability to traverse the BBB. Research suggests miRNA‐21 may impact pathways like RECK, TIMP3, and IGFBP3. It could improve glioma prognosis through minimally invasive miRNA‐based therapy (Beylerli et al. 2022). MiRNAs, epigenetic regulators, play a crucial role in cellular health and disease progression. Recent research has identified the link between miRNAs and molecular pathways linked to GBM pathophysiology (**Figure** **1**), including cell invasion, proliferation, and death. This finding could help determine potential therapeutic targets and explore multitargeted miRNA‐based treatments for GBM (Behrooz et al. 2023). Furthermore, a study demonstrates the development of ferroptosis, a cell death caused by iron‐dependent phospholipid peroxidation, and its impact on various cells. It investigates the xCT system, an amino acid antiporter that promotes glutathione synthesis and oxidative defense. Ferroptosis is particularly susceptible to brain tissue cancer cells, and its pharmacological control is crucial for drug‐resistant malignancies. The study identifies targets for triggering ferroptosis in cancer cells, including glutathione peroxidase 4 and the xCT amino acid antiporter (Nikolaev and Belopasov 2022).

## 4 Diagnosis of GBM

GBM is treated with chemotherapy, radiation, and surgical resection. Despite advancements in physiopathology, it still has a higher incidence, worse quality of life, and a median overall survival of 15 months (Gilard et al. 2021). GBM has a low prognosis, with a 4%–5% 5‐year survival rate and a 26%–33% 2‐year survival rate in trials. Current treatments prolong life without curing the disease, and adjuvant therapies such as immunotherapy and radiosensitizers do not reduce recurrence risks (Batash et al. 2017). The absence of efficient diagnostic techniques is one of the primary issues with GBM treatment. Current glioma diagnosis primarily relies on neurological examinations and neuroimaging techniques, which occur after disease progression (Posti et al. 2015; Mondal et al. 2017). The slow dispersion process of brain tumors, allowing structures to adapt to compression and deformation, is the primary reason for the late identification of GBM. Clinical manifestations may be absent even when clear morphological signs of tumor penetration into brain tissue are present (Sizoo et al. 2010). Antiangiogenic medications or chemoradiotherapy can significantly alter neuroimaging results, making follow‐up more challenging due to potential skewness (Peca et al. 2009). GBM treatment typically involves tumor removal surgery, followed by chemotherapy and radiation. However, these approaches often fail due to high relapse rates, tumor resistance, and significant neurological decline in patients (Ozdemir‐Kaynak et al. 2018). Early detection of GBM is essential due to the lack of focused medical therapy and the ineffectiveness of surgery in treating the condition. The current clinical procedures for GBM detection are often inefficient and rely solely on neuroimaging tests like MRIs. Brain tumors may not exhibit obvious clinical symptoms in their early stages, especially if they are not immediately apparent. Neuroimaging techniques are often underutilized for mass screening and do not always accurately indicate the presence or malignancy of a brain tumor (Aquino et al. 2017; Guillevin et al. 2014). The process of selecting a reliable analytical technique for analyzing potential biomarkers is also interconnected. Mass spectrometry is a valuable tool for studying biological materials due to its excellent performance, selectivity, sensitive detection, and ability to study various chemical components. Mass spectrometry techniques are becoming increasingly popular for laboratory diagnostics. Mass spectrometry detection techniques provide better indicators due to their improved detection limit, repeatability, accuracy, and precision when compared to antibody‐based methods (Gilbertson et al. 2015). Mass spectrometry techniques in proteomic analysis facilitate the identification of posttranslational modifications and tumor‐specific proteins, leading to promising outcomes. Protein expression patterns or changes in protein posttranslational modifications can be evaluated qualitatively or quantitatively and used as a diagnostic for CNS malignant tumors (J. Liu et al. 2014; Meier et al. 2018). Mass spectrometry‐based proteome analysis links disease states with protein changes, comparing healthy and diseased samples' proteomic profiles (Miyauchi et al. 2018). The integration of mass spectrometry into the standard clinical diagnosis of CNS oncological disorders depends heavily on this factor. Contemporary mass analyzers provide the ability to analyze multiple distinct molecular patterns indicative of various diseases in a single analytical cycle, facilitating routine application (Lenting et al. 2017). IDH mutations serve as favorable prognostic markers in adults, while CDKN2A deletions indicate high malignancy in IDH‐mutant astrocytomas. IDH‐wildtype astrocytomas may become GBMs under certain genetic changes. In pediatric patients, H3F3A mutations correlate with poorer prognosis, and MGMT promoter methylation is crucial for predicting responses to TMZ. Conversely, mismatch repair defects lead to a hypermutation phenotype associated with poor TMZ response. Liquid biopsies show promise for clinical use in diagnosis and prognosis (Śledzińska et al. 2021). Neuroimaging methods are essential for diagnosing GBM, which is subsequently confirmed through histological and molecular examinations. The biological behavior and clinical predictions of GBM are more effectively assessed through molecular analysis than traditional histopathology, especially when typical tumor hallmarks are absent. Liquid biopsy aims to detect and quantify tumor‐derived materials, such as nucleic acids and circulating tumor cells, from biofluids like blood and cerebrospinal fluid. This method presents a noninvasive alternative to surgical biopsies, potentially improving early recurrence detection and differentiating between tumor progression and pseudoprogression (Eibl and Schneemann 2023). A study investigated whether the methylation status of the MGMT promoter in GBM patients can be predicted using radiomic characteristics from multiparametric MRI. Analyzed were 277 GBM patients with segmented tumor subregions. Adding demographic variables did not improve performance. A weak correlation exists between imaging features and MGMT promoter methylation, demonstrating the necessity for further validation due to low diagnostic performance in the validation group (Doniselli et al. 2023). GBM may be analyzed for EGFR variant III (EGFRvIII) and EGFR amplification using a digital PCR (dPCR) technique with locked nucleic acid hydrolysis probes. This study included 62 patients, with the assay developed in an exploratory cohort of 19. The dPCR assay targets specific genomic regions to assess EGFR copy numbers and identify EGFRvIII. Validation with a cohort of 43 showed that the dPCR had 100% sensitivity and specificity in identifying EGFR amplification and concordance with RT‐PCR for EGFRvIII detection. The dPCR method's cost is significantly lower than fluorescence in situ hybridization. Overall, these findings suggest the feasibility of simultaneously detecting EGFR amplification and EGFRvIII in GBM using dPCR (Fontanilles et al. 2020). TERT promoter mutations C228T and C250T play a significant role in malignant transformation through telomerase activation, oncogenesis, and cell immortalization. In malignancies like GBM, mutations C228T and C250T are significant indicators, with their frequency reaching up to 80%. Promoter TERT mutations may interact with the rs2853669 SNP to affect GBM development and overall survival. Liquid biopsies enable the easy and precise identification of promoter TERT mutations, specifically C228T and C250T, along with other significant biomarkers. Liquid biopsy provides a promising approach for promoter TERT mutation analysis in personalized cancer treatment, particularly for brain cancer. This method evaluates the relationship between promoter TERT mutations and other biomarkers, demonstrating their clinical significance for early diagnosis, prognostication, and monitoring disease progression (Powter et al. 2021). IDH1 mutant GBM exhibit superior overall survival. A significant correlation was found between IDH1/p53 and improved median overall survival in cases with duplicate protein pairs. Additionally, individuals with IDH1/ATRX/p53 combinations also demonstrated superior overall survival. Thus, both individual and combined protein expression patterns are valuable for classifying GBM into prognostically significant subgroups (Meel et al. 2024).

## 5 Impact of Natural Compounds on Brain Cancer

### 5.1 Alkaloids

#### 5.1.1 Berberine

Berberine enhances the suppression of cancer cell migration in glioma cells mediated by As_2_O_3_, improving inhibition of glioma cell growth while decreasing invasion and motility. The combined administration can significantly reduce cancer cell metastasis (Lin et al. 2008). Additionally, berberine has a lower IC_50_ than temozolomide in GBM cells. The antitumor effect of this substance is primarily achieved through cellular senescence. It effectively inhibits EGFR, suppressing the RAF‐MEK‐ERK signaling pathway and preventing cellular senescence in GBM cells. Its potential use in treating GBM patients is suggested due to its mechanism of inhibition of EGFR, which is often elevated in the disease. It also significantly decreased the amount of EGFR in GBM cells (Q. Liu et al. 2015). Berberine has been studied for its potential in treating malignant brain tumors. In vitro experiments showed that berberine alone at a concentration of 150 µg/mL could kill 91% of malignant brain tumor cells. Combining berberine with BCNU at a concentration of 23 µM resulted in an average cell kill of 97%. In vivo treatment with berberine and BCNU showed cumulative cytotoxicity, and additional cytotoxicity was observed using a resistant 9L subline (R. Zhang et al. 1990). Furthermore, berberine nanoparticles have been investigated for potential glioma cell antitumor treatment due to their poor solubility and instability. TEM examination revealed the presence of berberine NPs in various solutions, and immunofluorescence analysis identified berberine‐Glu or berberine‐Water NPs. Berberine‐Glu NPs grew smaller and more uniform, and glioma cells readily absorbed them. It showed improved brain and glioma imaging in primary U87 glioma‐bearing mice. Berberine‐Glu NPs provide a promising approach to glioma precision treatment due to their improved stability and solubility (Y. Jin et al. 2022). A study explores the inhibitory effects of berberine on human glioma cells, controlling wtp53, mutp53, and their byproducts. It inhibits glioma cell proliferation, reduces cyclin D1, and may be a potential treatment (Z. Liu et al. 2020). Berberine and curcumin exhibit potent anti‐inflammatory and cancer‐fighting properties, with potential for more effective combination treatments, particularly in GBM cells, potentially slowing or stopping growth (Maiti et al. 2019). Moreover, berberine was tested for its antiangiogenic properties in human GBM. It prevented VEGFR2 and ERK from being phosphorylated, reducing angiogenesis in GBM xenografts (F. Jin et al. 2018). Berberine, a drug used to treat human glioma T98G cells, inhibited cell proliferation and led to cell death, causing an increase in G1 arrest. It upregulated P27 and downregulated CDK proteins, increasing apoptosis in T98G cells through procaspase activation and disruption of mitochondrial membrane potential (Eom et al. 2008). Another study found that Berberine significantly affects GBM cells' metabolic status, increasing autophagy flux and decreasing glycolytic capacity, leading to apoptotic cell death, decreased invasive characteristics, and limited proliferative potential, suggesting potential therapeutic benefits (Wang et al. 2016). Furthermore, berberine can decrease cellular viability and cause oncosis‐like death in gliomas by reducing oxygen consumption rate and activating autophagy. It suppresses phosphorylated extracellular regulatory protein kinases, preventing mitochondrial aerobic respiration and causing glycolysis. This leads to decreased metabolic activity and decreased energy generation efficiency in mitochondria (Y. Sun et al. 2018).

### 5.2 Terpenoids

#### 5.2.1 Lycopene

A randomized placebo‐controlled trial was conducted in a teaching hospital's radiation oncology department to evaluate the therapeutic effectiveness of lycopene in treating prostate cancer and high‐grade gliomas. Results showed nonsignificant differences in favor of lycopene, with higher time to progression, response at the last follow‐up, and overall response at 6 months. Nutritional supplements like lycopene may be beneficial for adjuvant care of high‐grade gliomas (Puri et al. 2010). Lycopene can penetrate the BBB, induce apoptosis, suppress proliferation, and control the cell cycle via ROS‐activated ERK pathways (Ko et al. 2024). Furthermore, a study investigated the effects of lycopene, gingerol, and silymarin on GBM cells. After 48 h, the viability of U118‐MG cells decreased significantly. These compounds may stimulate apoptosis and mitochondrial potential changes. Lycopene may serve as a potential treatment for GBM by targeting ROS‐induced cell growth and preventing cell death (Czarnik‐Kwaśniak et al. 2019). Moreover, another study investigates the impact of incorporating lycopene into chemotherapy and radiation therapy as an adjuvant treatment for high‐grade gliomas. Fifty patients with GBM or anaplastic astrocytoma suffered surgical excision but still had disease. They received radiation therapy at 60 Gy in 30 segments and concurrent weekly doses of 60 mg/m2 i/v Paclitaxel. Patients in two groups received radiation therapy and a placebo, with Group A showing better response rates and disease progression time, compared to Group B's 21‐week median (Puri et al. 2005).

### 5.3 Polyphenols

#### 5.3.1 Curcumin

**FIGURE 2:**
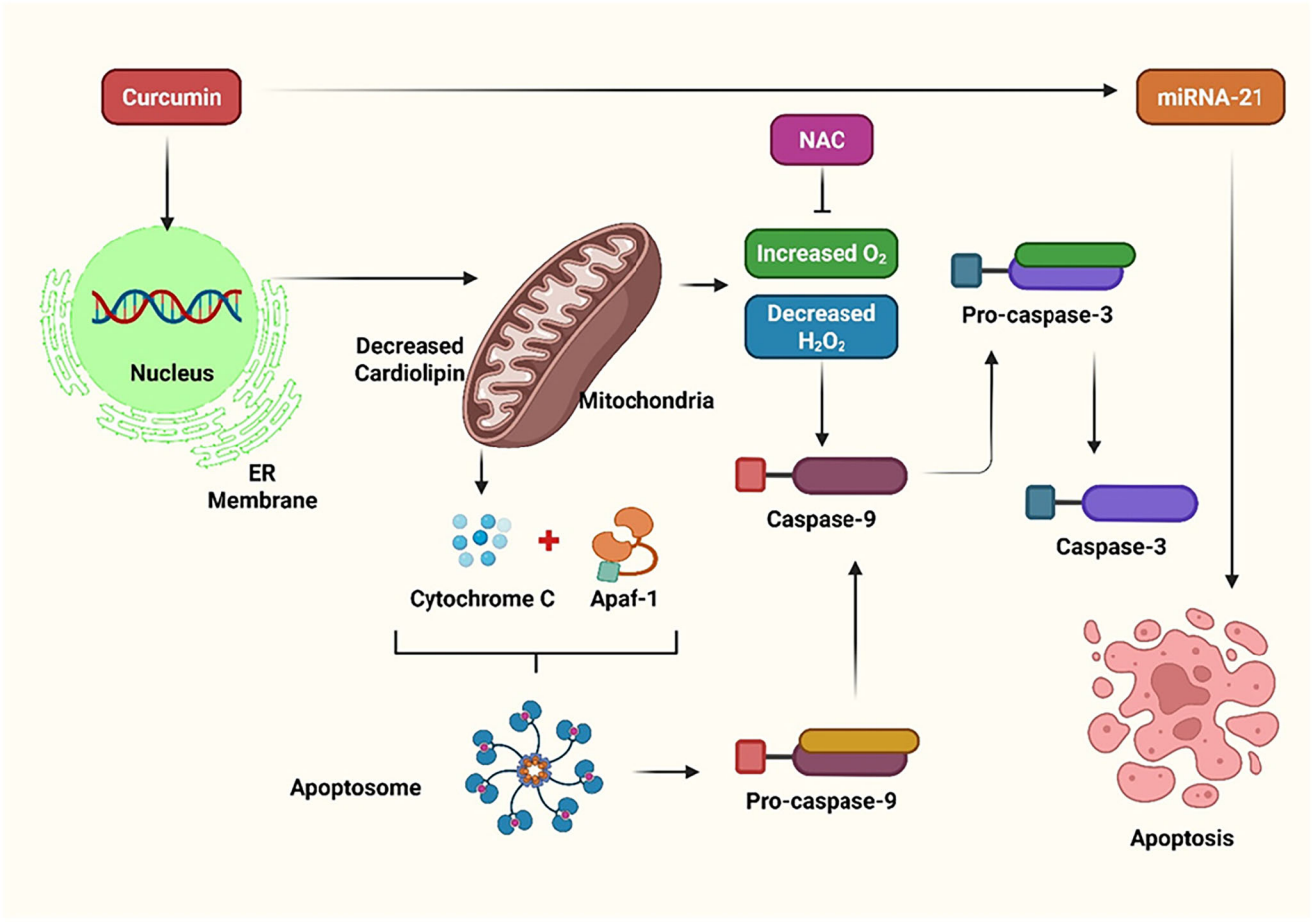
Curcumin causes apoptosis in human GBM cells by disrupting mitochondrial activity, causing DNA damage, and releasing cytochrome c. This leads to an apoptosome complex, triggered by cleaving and activating caspase‐3. Curcumin‐induced apoptosis is inhibited by NAC pretreatment, suggesting ROS involvement in cell death.

Curcumin, a polyphenolic component in turmeric, has been found to kill cancer cells in vitro. However, its anticancer action in the brain has not been investigated in vivo due to its poor water solubility. A soluble form of curcumin does not inhibit normal brain cell survival and crosses the BBB. In vitro studies showed that solubilized curcumin activates proapoptotic enzymes in mouse tumor cells, human oligodendroglioma, and lung carcinoma cells. Combining these findings could be a safe therapeutic approach for brain tumor treatment (Purkayastha et al. 2009). A study explores the use of curcumin in treating brain cancer using nanostructured lipid carriers (NLCs). NLC‐curcumin significantly improved curcumin's ability to target the brain and tumor, increasing its inhibitory efficiency from 19.5% to 82.3%. The FITC study confirmed that apoptosis was the primary mechanism for the inhibitory effect. The research demonstrated the therapeutic use of NLC‐curcumin in brain cancer treatment and provides a new approach to forming new anticancer drugs (Chen et al.^2016^). Curcumin is known for its anti‐inflammatory, antiproliferative, and antioxidant properties, which hold significant medicinal potential. It effectively treats GBM (**Figure** **2**) (Klinger and Mittal 2016). A study used curcumin and tyrphostins AG494 and AG1478, selective EGFR kinase inhibitors, to treat human brain cancer. The compounds significantly suppressed autocrine proliferation, cell cycle, and viability of LN229 cells, with curcumin enhancing their cytostatic and cytotoxic capabilities. The combination showed increased cytotoxic effect, decreased viability, apoptotic process activation, irreversible DNA damage, and decreased ROS in GBM cell culture (Bojko et al. 2015). Hossain et al. (2012) explored the synergistic effectiveness of paclitaxel and curcumin in treating GBM cells, including HBTSC. The combination of PTX and CCM reduced cell viability more efficiently than either medication alone. The combination therapy demonstrated promise as a treatment for regulating the proliferation of GBM cells (Hossain et al. 2012).

Moreover, Ostovar et al. (2023) demonstrated a promising nanocarrier for curcumin using a pH‐sensitive chitosan, gelatin, and carbon quantum dots nanocomposite. The nanocomposites showed increased cytotoxicity and a 22‐h half‐life, making them a promising biocompatible option for brain tumor treatment (Ostovar et al. 2023). Curcumin can supplement traditional and nontraditional treatments for brain tumors, reducing tumor development and preventing toxicity and resistance. Nano‐cargos can improve bioavailability and bioaccumulation. Clinical research suggests antibody‐conjugated nano‐curcumin combined with sub‐toxic doses of conventional medicines to improve outcomes by increasing tumor targeting, lowering therapy resistance, and preventing recurrence (Peter et al. 2022). Additionally, curcumin inhibits telomerase in brain cancer cells, triggering apoptosis and increasing cytotoxicity. Treatment with curcumin causes growth inhibition and cell cycle arrest in GBM and medulloblastoma cells. It upregulates PTEN genes, downregulates CCNE1, E2F1, and CDK2, and shortens telomeres by suppressing hTERT mRNA expression and telomerase activity (Khaw et al. 2013). Curcumin is a widely researched cancer treatment due to its strong anticancer effects. However, its short circulation half‐life and poor water solubility limit its use. The nanocarriers showed a persistent release profile, causing tumor cell death only and showing little harm to healthy cells. The addition of CeO_2_ NPs improved curcumin's therapeutic efficacy (Shabestari et al. 2025). A pH‐sensitive hydrogel for curcumin delivery achieved high efficiency and drug loading, extending curcumin release in acidic tumor‐like conditions, showing promise for targeted brain cancer treatment (Pourmadadi et al. 2025). Curcumin nanomicelles have antitumor effects on suppression of GBM. They inhibited U‐373 cell proliferation by altering the Wnt and NF‐κB pathways. The nanomicelles also led to apoptosis in cells after early G2/M cell cycle arrest. Curcumin nanomicelles effectively inhibit GBM cell invasion and growth by blocking Wnt/β‐catenin and NF‐κB pathways, thereby reducing cyclin D1 and p‐65 expression (Hesari et al. 2019). A dual‐targeted approach was developed using magnetic guidance and T7‐mediated active targeting delivery. The combination of medications (paclitaxel and curcumin) was co‐encapsulated into a T7‐modified magnetic NP system. This method showed higher efficacy in inhibiting tumor growth and improving brain delivery. In vivo investigations showed improved therapeutic efficacy and decreased side effects. This approach may enhance anti‐glioma treatment and brain drug delivery (Cui et al. 2016). Moreover, S. Luo et al. (2021) found that curcumin, bisdemethoxycurcumin, demethoxycurcumin, and dimethoxycurcumin promoted apoptosis, autophagy, ROS generation, and decreased cell viability, while dimethoxycurcumin inhibited cell motility and BCL‐2 (S. Luo et al. 2021). Curcumin's anticancer potential was demonstrated in vitro and in vivo using medulloblastoma models. It improved lifespan in mice, decreased tumor growth in xenografts, and may be a potential treatment (S. Lee et al. 2011).

#### 5.3.2 Resveratrol

**FIGURE 3:**
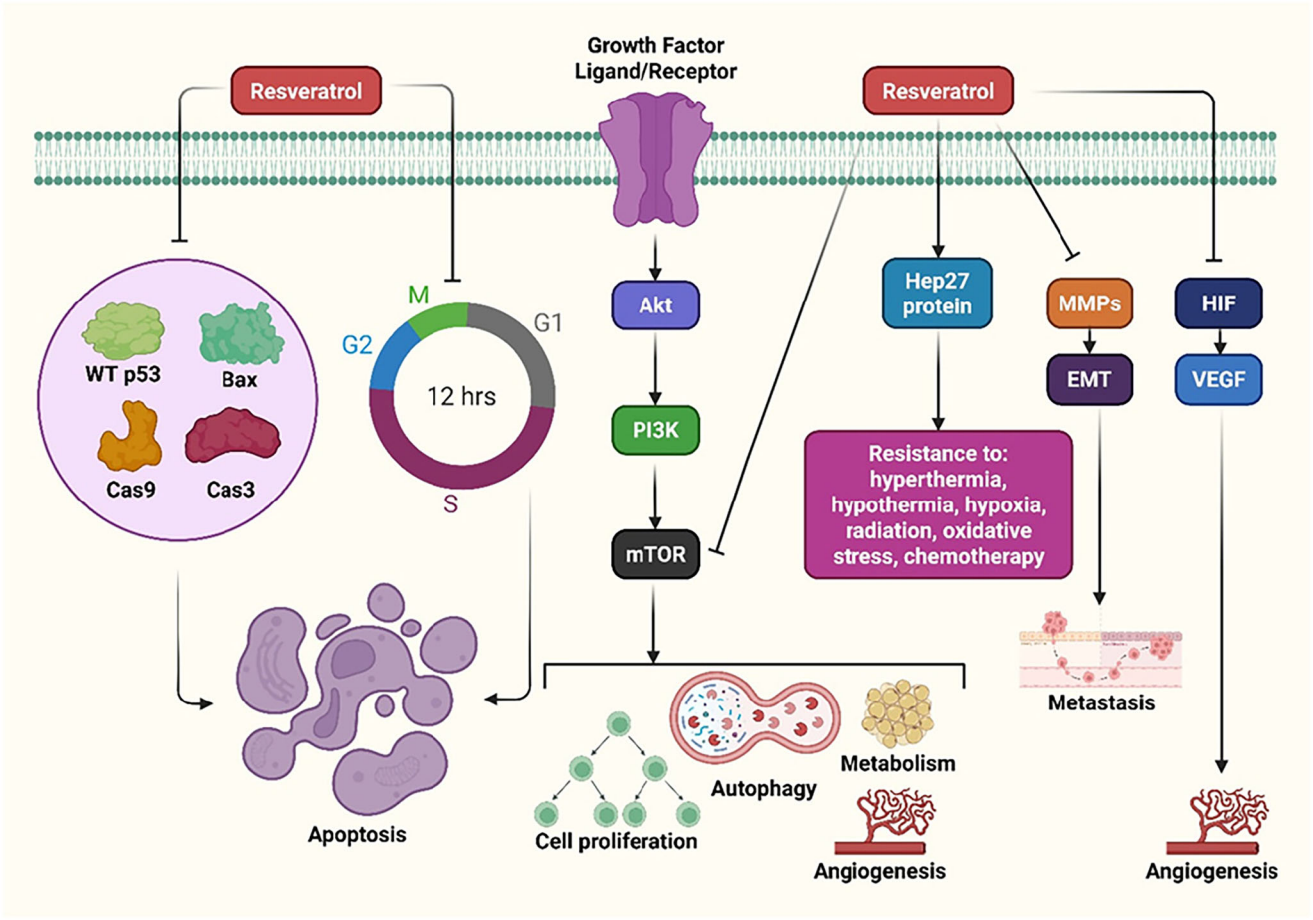
Demonstration of resveratrol, which prevents and treats glioblastoma.

Resveratrol has anti‐inflammatory, antioxidant, neuroprotective, and BBB‐crossing properties (Berman et al. 2017). IDHs mutant versions have been identified in brain cancer. NAD (+)‐dependent IDHs are essential for the Krebs cycle's NADH synthesis in mitochondria. Mutated IDH prevents glioma stem cell differentiation by increasing 2‐hydroxyglutaric acid, controlling VEGF, and increasing HIF‐1α, which enhances tumor microenvironment and induces glioma invasion. Resveratrol is found to maintain stable IDH levels in models of myocardial infarction and stroke. Additionally, it decreased NADH activity in liver cells and activated mitochondrial complex I. A phase I clinical investigation showed that treatment with resveratrol for 29 days resulted in low systemic levels of IGF‐1 and IGFBP‐3, indicating its antiproliferative activity (Kiskova et al. 2020). Additionally, resveratrol reduces TNF‐α‐induced invasion in U373MG glioma cells by controlling NF‐𝜅B activation (Desai and Bhushan 2017). Bose et al. (2020) found that postoperative resveratrol treatment significantly improves prognosis in advanced orthotopic GBM rats. It inhibits Src tyrosine kinase activity, which in turn prevents STAT3 action. It also inhibited STAT1 phosphorylation and JAK phosphorylation in human epidermoid carcinoma (A431) cells. It controls both the apoptotic pathway and caspase 3 activity (Bose et al. 2020). GBM patients' glioma cell lines were inhibited by altering the Wnt cascade, leading to reduced motility and increased cellular death (Song et al. 2018). Another study utilized resveratrol‐PLGA‐BNPs to enhance resveratrol's systemic circulation time and biological half‐life, demonstrating superior cytotoxicity against C6 glioma cells, improved cell internalization, and hemocompatibility. Resveratrol‐PLGA‐BNPs have superior anticancer activity against gliomas (Vijayakumar et al. 2016). Resveratrol demonstrated antiproliferative effects on GBM stem‐like cell lines and U87 glioma cells, inhibiting invasion, growth, and p53 expression, with intracranial injections increasing local drug concentration without side effects. It is a promising treatment option for genetically diverse tumors like GBM due to its ability to affect AKT and p53, among other anti‐tumorigenic pathways (Clark et al. 2016). Ibrahim et al. (2023) demonstrated the potential benefits of combining resveratrol and metformin in treating GBM. These decrease tumor development and volume in vivo, using both glucose‐independent and glucose‐dependent mechanisms. Both drugs inhibit cancer cell growth pathways, reducing proliferation and increasing apoptosis (Ibrahim et al. 2023). Resveratrol is used as an alternative treatment for GBM due to its resistance to traditional therapies and side effects (Arabzadeh et al. 2021). Moreover, another study explored resveratrol's (**Figure** **3**) impact on angiogenesis and anticancer effects in rat RT‐2 gliomas, revealing its cytotoxic effects, cell death, tumor reduction, increased survival, and prolonged survival time. However, it only affected intracerebral tumors at a higher dose, extending survival without changing survival rates. It decreased the proliferation of ECV304 cells in glioma cells (Tseng et al. 2004). Y. Zhang et al. (2023) found that resveratrol, which inhibits the PI3K pathway, may be beneficial in treating gliomas by inhibiting cell survival in a PTEN‐ and P53‐dependent manner. It also acts as a chemosensitizer, preventing cell proliferation and chemoresistance (Y. Zhang et al. 2023). A new multipurpose targeting carrier has been developed to treat brain tumors by integrating resveratrol into lipid bilayer membranes of multifunctional targeting liposomes. The liposomes showed significant inhibitory effects on brain tumor cells, with the greatest absorption and death observed in glioma cells (Kong et al. 2022). A study evaluated the synergistic efficacy of Soluplus polymeric nanoparticles loaded with paclitaxel and resveratrol against glioma cell lines. The paclitaxel and resveratrol‐loaded PNPs showed proper particle size, PDI, and % encapsulation effectiveness. They demonstrated synergistic antitumor activity against C6 glioma cells and increased bioavailability compared to pure paclitaxel and resveratrol. Combining paclitaxel and resveratrol PNPs could potentially increase anti‐glioma activity (Hussain et al. 2021). H. Jiang et al. (2005) investigated the molecular processes of resveratrol's anticancer effects on U251 gliomas. It causes dose‐ and time‐dependent mortality of U251 cells, enhances caspase‐3 activation, triggers caspase‐9 activation, and promotes proapoptotic Bax translocation. It also causes G0/G1 growth arrest and inhibits U251 proliferation. Several signaling pathways may be involved in resveratrol‐induced apoptotic death (**Table** **1**) (H. Jiang et al. 2005). Furthermore, a study found that resveratrol sensitivities of GBM cells vary due to sulfonation activity. LN229 cells were insensitive to resveratrol, while U251 cells were sensitive. Both cell lines had identical resveratrol metabolic pathways. The primary metabolite of human GBM cells is resveratrol monosulfate, produced by sulfotransferases. The inverse relationship between sulfotransferases‐mediated sulfonation activity and resveratrol sensitivity suggests that these factors could be crucial indicators for individualized resveratrol treatment (Z. Sun et al. 2012). Moreover, Castino et al. (2011) tested the anticancer potential of resveratrol using primary human GBM cultures and U87MG cells. Chronic resveratrol administration reduced cell migration and invasion, increased neuronal differentiation markers, and increased glial cell expression. Resveratrol can induce GBM cells to differentiate into glial and neuronal‐like cells, potentially aiding in chemotherapeutic treatment for astrocytomas (Castino et al. 2011).

**TABLE 1:** Natural compounds are used to prevent and treat glioblastoma.

Natural compounds	Cell lines	Finding	Ref.
Curcumin	A172 cells	In A172 human GBM cells, the potential effects of curcumin on autophagy, which controls curcumin‐mediated tumor cell death.	(J. Lee et al. 2019)
Resveratrol	U251 cells	Resveratrol causes U251 cells to die in a dose‐ and time‐dependent manner, as determined by tests for DNA fragmentation and lactate dehydrogenase release.	(H. Jiang et al. 2005)
Berberine	T98G cells	Treated human glioma T98G cells, inhibited cell proliferation and led to cell death, causing an increase in G1 arrest.	(Eom et al. 2008)
EGCG	U251	Reduced U251 glioma cell proliferation through laminin receptor, inhibiting invasiveness and causing apoptosis, influenced by the mitogen‐activated protein kinase pathway.	(H. Li et al. 2014)
Apigenin	U87 cells	Triggered ROS‐driven apoptosis, G2/M phase arrest, and caspase‐cascade pathway activation, significantly impacting U87 cells.	(Shendge et al. 2021)
Quercetin	U251 cells	Suppresses cell growth, apoptosis, and invasion in human GBM cells by downregulating MMP2 and MMP9 levels, affecting cell survival, migration, and cycle progression.	(Y. Liu et al. 2017)
Luteolin	U‐87 MG and T98G cells	Inhibited GBM cell invasion and migration in U‐87 MG and T98G cells.	(Cheng et al. 2013)
Lycopene	U‐118MG cells	Demonstrated a substantial decrease in the viability of GBM cells, U118‐MG.	(Czarnik‐Kwaśniak et al. 2019)
Baicalein	U251 cells	Reduced GBM U251 cell viability and apoptosis, with synergistic effects observed when BA and an NF‐κB‐P65 inhibitor were used.	(G. Jiang et al. 2016)
Genistein	C6 cells	Inhibited the enhanced mitochondrial ROS accumulation in C6 cells induced by Aβ.	(Ma et al. 2013)
Chrysin	C6 cells	Decreased the activity of CDK2 and CDK4 kinases, suppressed proteasome activity, and enhanced p38‐MAPK activation.	(Weng et al. 2005)
Daidzein	Glioma cells	Inhibited Bcl‐2, which reduces apoptosis caused by Daidzein and TRAIL.	(Siegelin et al. 2009)

#### 5.3.3 Epigallocatechin‐3‐Gallate

In vitro, EGCG can stop the growth of glioma cells and other human cancer cell types. It affects glioma cell invasiveness, apoptosis, and cell proliferation. It also inhibits cell proliferation by interfering with the PDGF pathway (Sharma et al. 2012). The PDGF‐Rb receptor, which attaches to the PDGF‐BB ligand, transmits mitogenic signals by autophosphorylating tyrosine residues (Westermark et al. 1995). Glioma development and progression are linked to the precise autocrine activation of PDGF‐Rb. When EGCG (50 µM) was administered to GBM A172 cells. A172 cell spheroid formation was suppressed when PDGF‐Rb was blocked (Sachinidis et al. 2000). EGCG inhibits PDGF‐Rb phosphorylation, thereby suppressing PDGF‐induced proliferation in various cancer types. It directly interacts with PDFG‐BB, inhibiting its binding to PDGF‐Rb (Sakata et al. 2004). Additionally, EGCG prevents PDGF‐Rb phosphorylation in glioma cells by preventing PDFG‐BB from attaching to its receptor. The experiments frequently utilize tetrazolium compounds like MTS, MTT, and WST‐8 due to their conversion into purple formazans by live cells. Moreover, ATP quantification techniques can be used to evaluate cell viability (Le et al. 2018). The tumorigenic cell population in GBM comprises these cells that can proliferate indefinitely, aiding tumor formation and maintenance (Hira et al. 2015). A medication can induce cell death through its cytotoxic effects. Dead cells can be detected using various tests like Wright's stain, propidium iodide, trypan blue, TUNEL, and annexin‐V (Elmore 2007). By inhibiting MT1‐MMP, EGCG can prevent glioma cells from becoming invasive (Yokoyama et al. 2001). Through gene silencing, EGCG lowers MT1‐MMP expression. When pro‐MMP2 in MMP2 is activated, MT1‐MMP encourages glioma cell invasion. Gelatin zymography revealed that EGCG significantly reduced MMP2 activity in U87 in a dose‐dependent manner. EGCG was found to deactivate pro‐MMP2 in U87 cells via MT1‐MMP, even after exposure to concanavalin A, a potent inducer of pro‐MMP2 production. EGCG inhibits MMP2 activation through MT1‐MMP, reducing glioma cell invasiveness due to MMP2 being a key protease involved in cell invasive behavior (Demeule et al. 2000; Annabi et al. 2002; Mook et al. 2004). ROCK activation increases RhoA expression by phosphorylating CD44's cytoplasmic domain and promoting CD44/HA binding. EGCG reduces CD44 cleavage by inhibiting MT1‐MMP expression and activity, thus reducing the production of HA (Annabi et al. 2005). Additionally, EGCG inhibits U87 cell invasiveness by reducing F‐actin levels after disrupting the design of the cytoskeleton. EGCG's suppression of MT1‐MMP can disrupt the actin network, as it influences the actin cytoskeleton's topology via the MAPK pathway, promoting cell migration (Agarwal et al. 2008). Yokoyama et al. (2001) investigated the effects of EGCG on rat glioma cell lines C6, U‐373 MG, U‐87 MG, and MtT/E. Results showed significant reductions in cell viability at various doses, with EGCG significantly decreasing C6 cell viability. The highest dose caused apoptosis in MtT/E cells. EGCG inhibits malignant brain tumors and may be mediated through IGF‐I (Yokoyama et al. 2001).

Another study explores the clinical use of EGCG on gliomas. It restricts invasion, stops proliferation, and causes cell death in glioma cell lines. It also decreases the effect of bortezomib and increases the effectiveness of anti‐glioma treatments. Its mechanisms of action include inhibiting pro‐survival proteins, blocking metalloproteinases, cytokines, and chemokines, and disrupting redox state and metabolism (Le et al. 2018). Furthermore, a study utilized EGCG, TRAIL, or a combination of both to treat three GBM cell lines (U87, A172, and U251). EGCG and TRAIL, when combined, can potentially offer an effective treatment for glioma cells that are resistant to TRAIL, causing rapid cell death (Siegelin et al. 2008). EGCG can enter the brain through oral ingestion and potentially act as an anti‐tumoral drug. It can selectively inhibit DAOY cell migration on collagen, targeting the invasive nature of medulloblastomas (Pilorget et al. 2003). GBM treatment has been limited by single‐drug therapy and inadequate targeting. The nanoreactor, which disintegrates, allows precise release of DOX, Fe^3+^, and EGCG into tumor cells. The nanoreactor's in vivo studies showed that sustained release of DOX and Fe^3+^ produced a unique therapeutic effect, and it shows promise for future treatment of GL261 GBM. The first‐ever HA‐EGCG coating was formed using a quick method, utilizing a metal‐tea polyphenol network coated with HA‐EGCG (Mu et al. 2021). Moreover, EGCG can enhance the therapeutic efficacy of temozolomide in GBM preclinical models. Animals treated with temozolomide exhibited higher levels of GRP78 expression, while those treated with EGCG showed lower levels (T. Chen et al. 2011). McLaughlin et al. (2006) investigated the potential of EGCG in altering the response of GBMs to ionizing radiation (IR). The researchers transfected cDNAs encoding Survivin, RhoA, or Caveolin‐1 into U‐87 human GBM cells and administered sublethal radiation. IR decreased cell proliferation by 40%, while Survivin overexpression produced cytoprotective effects. EGCG‐pretreated cells showed increased IR sensitivity and decreased RhoA expression (McLaughlin et al. 2006). Another study found that metformin and EGCG can effectively treat glioma by enhancing cell apoptosis and suppressing cell growth, while increasing OS‐induced cell death. TMZ, MET, and EGCG together could improve GBM patients' clinical results (Kuduvalli et al. 2021).

#### 5.3.4 Apigenin

Apigenin and acacetin have anticancer effects on GBM cells (U87). These significantly impact U87 cells, with ROS generated by acacetin and apigenin causing cell cycle arrest and caspase‐cascade pathway activation (Shendge et al. 2021). In vitro, apigenin has antitumoral effects on a variety of tumors. Apigenin, by altering the production of TNF and IL‐10, simultaneously enhances the proinflammatory M1 microglia profile (Coelho et al. 2019). Treatment with this flavone enhances tumor immune response and significantly increases nitric oxide generation, known for its inflammatory properties (Coelho et al. 2016). Apigenin exhibits strong anti‐inflammatory properties in C6 glioma cells activated by lipopolysaccharide and IFN‐γ, suppressing iNOS expression and inhibiting cytokine release (Mazzio et al. 2017). The observed difference may be due to variations in the concentrations of the phytochemical or different cellular environment conditions. The concentration range of apigenin that decreases glioma cell viability is broad, ranging from 1 to over 30 µg/mL. Unfortunately, if cells are exposed to culture conditions lacking apigenin, the tumor‐suppressive action can be reversed (Coelho et al. 2016; Wang et al. 2013). Apigenin controls GBM cells by inducing apoptosis, selectively controlling them while leaving normal astrocytes unaffected. It inhibits Akt, Bcl‐2, MAPK, and mTOR, potentially having antitumor properties by promoting cell growth, proliferation, differentiation, and migration (Das et al. 2010). Apigenin is a protein that inhibits the activity of CK2, a key enzyme in the growth of U87 glioma cells (Ahn et al. 2006). Additionally, apigenin not only promotes apoptosis but also enhances autophagy by decreasing p62 levels and upregulating its indicators like LC3‐II and beclin‐1 (I. Jeremic et al. 2013).

A study found that C8‐prenylated apigenin derivatives can produce even better results in terms of inducing apoptosis, primarily through their actions on caspase‐3 and caspase‐7 (Wätjen et al. 2007). Apigenin inhibits the release of TGF‐β1 and VEGF, which are crucial for the growth of neoplasms, including U343 and U118 gliomas (Freitas et al. 2011; Schindler and Mentlein 2006). MiRs, which regulate fundamental metabolic processes, are gaining increasing attention for their role in various illnesses, including cancer. The antitumoral potency of apigenin is likely due to its ability to alter the expression of these molecules. The compounds significantly boost tumor‐suppressive miRs, reducing invasion and enhancing glioma cell death (Chen et al. 2016). Apigenin may aid in the formation of the ECM by promoting the synthesis of fibronectin and preventing its breakdown (Santos et al. 2015). GBM stem cells are identified as a significant factor in the formation, treatment resistance, and recurrence of tumors. Targeting c‐Met signaling to eliminate GBM stem cells may improve the prognosis of GBM patients by preserving their stem‐like phenotype. Apigenin treatment significantly reduced the invasiveness and self‐renewal potential of GBM stem‐like cells (B. Kim et al. 2016; Feng et al. 2012). Apigenin, a medicinal drug that increases radiation sensitivity in gliomas, is a promising option for radiotherapy due to its potential benefits. Radiosensitization in culture mediums may be due to decreased HIF‐1α expression, leading to downregulation of glucose transporter, NF‐κβ, and PKM2 (Zhao et al. 2021). Chemotherapy, with TMZ being the current gold standard, is a highly regarded method for treating gliomas alongside radiotherapy. The study suggests that in in vitro experiments, a combination of apigenin and TMZ treatment may yield better outcomes. The combination of two medications effectively inhibits glioma cell growth and causes their arrest at the G2 phase, outperforming their use alone (D. Wang et al. 2024). Apigenin can work alongside other phytochemicals extensively studied for potential use in glioma therapy approaches (Wang et al. 2013; L. Chen et al. 2021). Furthermore, apigenin injections were found to be ineffective in reducing tumor size or vascularity, with only marginal effects on the mean size of slowly growing C6 gliomas in mice. It reduced angiogenesis but not tumor development, possibly due to increased matrix‐degrading enzymes, dose insufficiency, or deterioration of nonspecific immune defense due to its anti‐inflammatory effects (Engelmann et al. 2002). Moreover, a study on mice showed that a 20 mg/kg dose of apigenin, a radiotherapy‐sensitizing drug, significantly increased the volume of glioma in animals exposed to 8 Gray irradiation. The reduction in lactic acid accumulation, a key factor in radio resistance, is a result of this (C. Jia et al. 2022).

#### 5.3.5 Quercetin

Quercetin is known to downregulate PI3K activity. It decreased Akt protein in U87 and U251 GBM cells, enhancing the effectiveness of TMZ treatment compared to chemoradiotherapy by downregulating PI3K/Akt activity (Pozsgai et al. 2013). Quercetin's docking into the PI3K site validates its PI3K blocking. It triggers apoptosis in human GBM T98G cells by activating signaling pathways leading to mitochondrial death. It activates FADD, caspases 8, 10, and 3, causing apoptosis. Exposure to quercetin activates caspases 3 and 9, releasing cytochrome C from mitochondria (Atiq and Parhar 2020). HSF, which is involved in mitochondrial apoptosis, is blocked by quercetin (Jakubowicz‐Gil et al. 2013). By suppressing the apoptosis‐inhibiting protein survivin, quercetin makes GBM cells more sensitive (Siegelin et al. 2009). Cell viability loss is avoided by quercetin and caspase blockers, which increase caspase. Quercetin, by downregulating survivin and ERK, inhibits survivin and antiapoptotic protein production, indicating it functions via a caspase‐dependent route to cause GBM cell death (E. Kim et al. 2008). Since phospholipase D (PLD) is a cancer regulator, inhibiting it may be advantageous in the pathophysiology of GBM regulated by PLD (Mathews et al. 2015). Quercetin inhibits the expression of PLD1. Furthermore, quercetin, by inhibiting NF‐κB transactivation, decreases the expression of PLD1 linked to NF‐κB (Park and Min 2011). A poor prognosis was typically projected by elevated HSP expression in malignancies, which also increased resistance to treatment approaches. Increased HSP expression in transformed cells plays a crucial part in suppressing apoptosis, which results in treatment resistance and tumor growth (Ciocca et al. 2003). HSF1 hyperactivation reinforces the high amounts of HSP in the cancer cell, facilitating invasion and metastasis. Quercetin has demonstrated anticancer action by targeting HSF1‐dependent HSPs in a variety of cancer cell lines (Park and Min 2011). Overexpression of HSP27 can reduce apoptotic cell death, promote carcinogenesis, and enhance multidrug resistance by directly interacting with various apoptotic proteins (Z. Yu et al. 2010). Additionally, quercetin showed stronger cytotoxic action when coupled with gemcitabine or cisplatin (Hsu et al. 2011). IL‐6, an inflammatory cytokine linked to cancer, controls STAT3 activity and is elevated in GBM. JAK and STAT3 activation were decreased by quercetin (Michaud‐Levesque et al. 2012). Quercetin induces cell cycle arrest in human glioma cell lines, apoptosis, and a reduction in proliferation (Braganhol et al. 2006). Furthermore, Quercetin overexpresses cleaved PARP and increases caspase‐3, ‐7, and ‐9 in GBM cells, causing apoptosis linked to oxidative and ER stress (Kusaczuk et al. 2022; Kusaczuk et al. 2024). Additionally, quercetin suppresses the production of two ECM proteins linked to the development of gliomas: fibronectin and MMP‐9 (Pan et al. 2015). Quercetin impacts the overexpression of the anti‐apoptotic chaperone protein, Hsp72, which is linked to tumor chemoresistance in tumor cells. It triggers Hsp72 to enter the nucleus in anaplastic astrocytoma cells, potentially enhancing cell survival and chemoresistance (Jakubowicz‐Gil et al. 2014; Jakubowicz‐Gil et al. 2011). When combined with temozolomide and sorafenib, quercetin is more effective against astrocytoma and GBM when Hsp72 is silenced with certain siRNAs (Jakubowicz‐Gil et al. 2013). Quercetin, when esterified and brominated, produces derivatives that are more selective for glioma cells and have a greater cytotoxic effect than quercetin alone (Dell'Albani et al. 2017). Moreover, quercetin treatment inhibits lipid synthesis enzymes in glioma cells, potentially due to its ability to decrease membraneogenesis, a crucial process for efficient lipid production (Damiano et al. 2019). Furthermore, quercetin and rutin altered microglial cells' inflammatory response, increasing IL‐1β, IL‐18, and TNF expression while reducing carcinogenic IL‐6 levels. The flavonoids prevented the neoplastic cells from proliferating and migrating in microglia/glioma cocultures (da Silva et al. 2020). Quercetin inhibits the GSK‐3β/β‐catenin pathway, which in turn downregulates the ZEB1 transcription factor. This transcription factor is crucial for EMT, enabling glioma cells to become more motile, invasive, and metastasizing (Wang et al. 2016). Additionally, quercetin has been investigated in combination with other compounds that might have a synergistic effect on GBM. Quercetin and sodium butyrate, inhibitors of histone deacetylase, prevent protective autophagy, a breakdown mechanism that allows tumor cells to survive despite malnutrition or cancer treatments. The synergistic activity of quercetin and sodium butyrate led to the apoptosis of both rat and human GBM cells. Overcoming resistance to chemotherapeutics like temozolomide may be aided by this action (Taylor et al. 2019). The development of a quercetin‐losartan hybrid molecule, which utilizes quercetin's antioxidant properties and losartan's AT1R angiotensin receptor suppression, could be a promising advancement (Tsiailanis et al. 2020). Researchers are developing various nanoformulations to discover effective medication delivery methods for clinical use. Magnetoliposomes loaded with quercetin and freeze‐dried nanomicelles effectively decrease the viability of C6 glioma cells in vitro (Wang et al. 2016; Dos Santos et al. 2019).

Ersoz et al. (2020) used a polymer of a lactic acid derivative loaded with quercetin to form four nanoparticles of different sizes. Researchers discovered that the smallest particle, measuring 215.2 nm and containing 25 mg of quercetin, was most effective in penetrating glioma cells and exhibiting antitumoral effects (Ersoz et al. 2020). Another study developed nanoliposomes containing quercetin and 3‐bromopyruvate (3‐BP), a potent alkylating substance with cytotoxic activity against various cancer cells by depleting ATP. This formulation has demonstrated efficacy in preventing the formation of astrocytomas in vitro (Soriano‐Ursúa et al. 2024). Moreover, a study of in vivo research on quercetin's impact on gliomas indicates that a degree of prudence should be exercised before practical implementation. The size of C6 gliomas in a rat model was increased, contradicting the in vitro results. The study found a slight decrease in systemic T‐cell proliferation and lymphocyte infiltration into the tumor, suggesting a compromised immune response (Zamin et al. 2014). The brain tissue examination revealed quercetin concentrations of 0.16 µg/g (∼530 nM), significantly lower than the in vitro concentrations used (Zamin et al. 2009). Quercetin‐treated mice showed a lower weight loss compared to TMZ‐treated mice, indicating a low toxicity profile in glioma treatment (Chen et al. 2022). Nanoliposomes and freeze‐dried quercetin‐loaded nanomicelles are being developed to improve the physicochemical properties of quercetin and its efficacy in drug delivery systems. Both strategies effectively increase phytochemicals in glioma‐bearing mice's tumor tissue, prolonging their lifespan and decreasing tumor size (Wang et al. 2016; Soriano‐Ursúa et al. 2024). Quercetin‐based nanoparticles stabilized with amphiphilic polymers are 18 times more effective in reaching cancerous CNS structures than in brains free of tumors. They significantly disrupt tumor vasculature development in mouse GL261 syngeneic glioma models and human PS30 GBM xenografts by inhibiting VEGFR2 activity (F. Liu et al. 2022).

#### 5.3.6 Luteolin

Luteolin is a highly abundant compound found in various vegetables such as carrots, parsley, and celery (Z. Chen et al. 2012). Lutein, through its ROS/ER stress pathway and mitochondrial dysfunction, may inhibit cell growth, trigger apoptosis, and decrease iNOS expression, potentially leading to glioma cell death (Q. Wang et al. 2017). By upregulating miR‐7‐1‐3p, luteolin causes cell death (Chakrabarti and Ray 2016). Additionally, it inhibits the growth of glioma cells by downregulating the expression of EGFR mRNA (Anson et al. 2018). Additionally, these substances suppressed the expression of PKCα, iNOS, and XIAP and reduced angiogenesis events by inducing apoptosis (Markaverich et al. 2010). Luteolin activated glucose‐associated protein and caspase‐3 while blocking p‐AKT. IL‐1β caused these processes, which led to increased nuclear translocation of NF‐κB (Zhou et al. 2009). Additionally, luteolin decreases proliferation by regulating the Akt and MAPK signaling pathways. The excessive activity of EGFR in GBM is due to its overexpression (Anson et al. 2018). Luteolin and valproic acid, a traditional anticonvulsant medication, effectively suppress the Akt pathway (Han et al. 2021). Moreover, luteolin and erlotinib, an EGFR inhibitor, have been studied in clinical trials for their synergistic antiproliferative effect in treating GBM (Powe et al. 2022). The production of OS and mitochondrial dysfunction is also responsible for luteolin's antiproliferative effects (Q. Wang et al. 2017). Luteolin induces dysplastic cell death via the extrinsic apoptosis pathway, increasing PARP, caspase‐3, and caspase‐8 levels and upregulating tumor suppressor miRNA expression, suggesting antiproliferative properties against gliomas (You et al. 2019; Sejda et al. 2022). Furthermore, traditional chemotherapeutics like temozolomide and carmustine are less effective than the synergistic anti‐glioma activity of luteolin and silibinin (Chakrabarti and Ray 2015).

Additionally, luteolin triggers autophagy in GBM cells, potentially enhancing cell survival under specific circumstances. Autophagy induction decreases the antiproliferative effect of luteolin by inhibiting its ability to induce apoptosis. The autophagy inhibitor 3‐methyladenine effectively increased luteolin‐induced apoptosis in GBM, suggesting a potential combination approach (H. Lee et al. 2021). Luteolin affects S1P synthesis, a cancer biomarker, by altering the equilibrium between S1P and ceramide, potentially affecting tumor genesis and invasion (Navone et al. 2023). Furthermore, luteolin induces apoptosis in GBM cells in xenograft‐bearing mice, reducing tumor volume without weight loss or hepatotoxicity, due to ROS generation, mitochondrial dysfunction, ER stress, and increased caspase activity (Q. Wang et al. 2017). Zheng et al. found that nanomicelles containing this polyphenol were used to improve the low bioavailability of luteolins. The molecule's increased water solubility, prolonged plasma presence, and enhanced antitumoral efficacy in mice with GBM are enhanced (S. Zheng et al. 2017). A novel approach utilizes nanoformulations of luteolin combined with folic acid, targeting folate receptors, to improve its antiproliferative effects on gliomas in mice (Wu et al. 2019).

#### 5.3.7 Baicalein

Baicalein was administered at different doses to U251 glioma cells for varying durations (12, 24, 36, or 48 h). Baicalein decreased the viability and clonality of U251 cells, altered cell cycle distribution, and increased apoptosis. It downregulated MMP‐2 and MMP‐9 levels while activating the PI3K/Akt pathway, leading to cell cycle arrest in the G0/G1 phase. LY294002 countered the inhibitory effects of baicalein on MMP levels and invasiveness in gliomas, whereas IGF‐1 elevated these factors. By activating the PI3K/Akt pathway, baicalein therapy can inhibit glioma cell invasion and cause apoptosis (Yang et al. 2021). A study investigated baicalein's impact on cisplatin‐induced cell death in human glioma cells. It inhibited cisplatin‐induced apoptosis and cell viability loss in a dose‐dependent manner, while maintaining cell viability. It also prevented cisplatin‐induced caspase activation, mitochondrial depolarization, cytochrome c release, and Bax expression, thus preventing cisplatin‐induced death (S. Lee et al. 2005). Researchers evaluated baicalein's antitumor efficacy using orthotopic glioma models. It significantly suppressed tumor growth and prolonged survival in mice with U87 gliomas, inhibiting cell growth, increasing apoptosis, attenuating tumor and brain edema, and reducing HIF‐1α, VEGF, and VEGFR2 expression (F. Wang and Jiang 2015). Furthermore, a study investigates the role of baicalein and LGR4 in brain gliomas. It is associated with poor prognosis and is significantly expressed in brain gliomas. LGR4 knockdown and overexpression led to apoptosis and suppressed EGFR phosphorylation and proliferation. Baicalein and an EGFR inhibitor reversed these effects. Baicalein, by downregulating the LGR4‐EGFR pathway, reduced malignant behavior and induced apoptosis (Zhang et al. 2024). Jiang et al. investigated the impact of baicalein on the human GBM U251 cell line, a malignant type of cerebral glioma with a poor prognosis. Baicalein treatment significantly reduced U251 cell viability and induced apoptosis, with NF‐κB‐p65 activity being significantly suppressed. When baicalein and an NF‐κB‐P65 inhibitor were combined, Bcl‐2 expression decreased, Bax and cleaved‐caspase3 expression increased, and the viability of U251 cells was suppressed (**Table** **1**) (G. Jiang et al. 2016). Moreover, baicalein inhibits cancerous tumor invasion and growth in vitro and in vivo. This study investigated its antitumor actions in GBM using U251 cells. Baicalein significantly decreased cell migration and proliferation. EGF‐induced tumor proliferation and migration are caused by EGFR/Akt pathways. It reduced EGFR and Akt phosphorylation (Y. Wang et al. 2019).

#### 5.3.8 Genistein

Genistein, which is specifically present in *Psoralea corylifolia* and *Pueraria lobata*, is 5,7‐dihydroxy‐3‐(4‐hydroxyphenyl) chromen‐4‐one (Kikuta 2020). Moreover, autophagy activation plays a critical part in genistein's chemopreventive pathway against a variety of tumor forms (Kikuchi et al. 2019). Furthermore, genistein was found to cause growth arrest in A172 and ONS76 radiosensitive GBM cells, but not in radioresistant cells. Genistein has a chemopreventive effect rather than cytotoxic potential, and as such, it may be a useful part of a combined chemotherapeutic regimen (Trachootham et al. 2009). In glioma cell lines, including U87 and C6, genistein and carmustine were found to have synergistic growth‐inhibitory action in prior work (Khoshyomn et al. 2002). By inhibiting topoisomerase II and causing G2/M phase arrest, genistein produces cytotoxicity in LN308, LN18, and T98G GBM cells while upregulating tumor suppressor genes such as p21 (Schmidt et al. 2008). Additionally, genistein inhibits telomerase reverse transcriptase transcriptional activities, which are essential for encoding the telomerase catalytic site in GBM and other neuroblastoma cells. By blocking urokinase plasminogen activator, which is involved in several activities of the metastatic cascade, and tyrosine kinase EGFR suppression, genistein prevents the growth of tumor cell invasion (Khaw et al. 2012). Another well‐known inhibitor of protein tyrosine kinase is genistein, which binds to the tyrosine kinase domain in competition with ATP to prevent the activation of downstream signaling pathways mediated by tyrosine kinase (Sarkar et al. 2009).

#### 5.3.9 Chrysin

**FIGURE 4:**
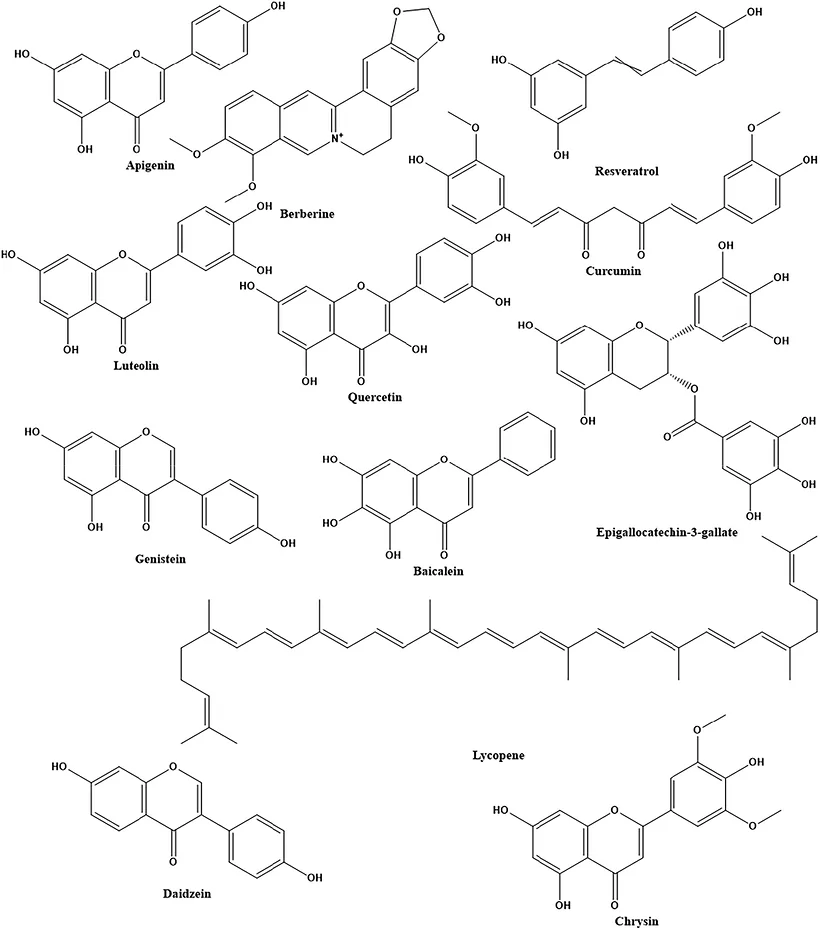
Chemical structures of natural compounds which are used for treating glioblastoma.

Chrysin (**Figure** **4**) is a flavonoid derived from *Passiflora incarnata* and *Passiflora caerulea* (Naz et al. 2019). Additionally, chrysin induces G1 cell cycle arrest in C6 glioma cells by inhibiting proteasome activity or stimulating the p38/MAPK pathway, leading to p21Waf1/Cip1 protein aggregation (Weng et al. 2005). Additionally, chrysin suppresses tumor invasion and migration in GBM cell lines by downregulating the ErK/Nrf2 pathway. In anaplastic gliomas, chrysin inhibits Nrf2, which in turn inhibits the production of heme oxygenase‐1 and NADPH quinine oxidoreductase‐1 (J. Wang et al. 2018). Furthermore, in the mouse model, chrysin improves the activities of glutathione peroxidase, superoxide dismutase, and catalase while decreasing ROS (Souza et al. 2015). Acute promyelocytic leukemia responded well to the combination of chrysin and silibinin, whereas GBM exhibited poor sensitivity (Liao et al. 2014). By inhibiting the PI3K‐Akt and ERK pathways, chrysin reduces Nrf2 expression at the mRNA and protein levels, which lowers anticancer treatment resistance (Gao et al. 2013). Chrysin causes apoptosis, which is linked to Akt dephosphorylation in the PI3K signaling pathway (Khoo et al. 2010). Moreover, chrysin's acute metabolism in humans results in decreased bioavailability despite its greater therapeutic benefits. Chrysin's poor oral bioavailability is demonstrated by the strong affinity of its metabolizing enzymes (Mani and Natesan 2018).

#### 5.3.10 Daidzein

Soy isoflavone daidzein has potential for cancer treatment. It treats GBM cells and human astrocytes. It also altered the intrinsic apoptotic pathway, increasing caspase‐9 activation. However, it inhibited Bcl‐2, which reduces apoptosis caused by daidzein and TRAIL. Bcl‐2 plays a crucial role in TRAIL‐daidzein‐mediated malignant glioma cell death (Siegelin et al. 2009). A study investigated the anticancer effects of daidzein against GBM using network pharmacology, molecular docking, and in vitro evaluation. The research aimed to identify molecular targets, analyze signaling pathways, and assess cytotoxic and antioxidant effects on brain tissues and cells. Daidzein showed significant binding affinity to key targets. In vitro tests showed reduced OS, increased SOD and GSH, and decreased cell viability. Daidzein could be a potential neuroprotective or adjuvant therapy for GBM (Praisthy L J et al. 2025). Zhang et al. found that daid002, a new daidzein derivative, inhibited the growth of GBM multiforme by inhibiting the interaction between moesin and CD44 on the cell membrane, thereby reducing moesin phosphorylation and cyclin D1 expression, and significantly inhibiting GBM in vivo (X. Zhang et al. 2018).

## 6 Crosstalk of Signaling Pathways

In T98G glioma cells, gartanin inhibited migration, proliferation, and colony formation in a concentration‐ and time‐dependent manner, without affecting mouse normal neuronal HT22 cells. It induced G1 phase cell cycle arrest, increased p27Kip1 expression, and decreased cyclin D1 expression. Gartanin also significantly reduced the secretion and activity of MMP‐2/‐9, likely through modulation of the MAPK signaling pathway. Furthermore, it enhanced autophagy in T98G cells, shown by increased GFP‐LC3 fluorescence, elevated Beclin 1 and LC3‐II expression, and reduced p62 levels. This was linked to the inhibition of the PI3K/Akt/mTOR pathway. Autophagy inhibitors 3‐methyladenine and chloroquine reduced gartanin's effects on cell viability. Gartanin demonstrated an antiproliferative effect through cell cycle arrest mediated by autophagy, and its anti‐migratory effect is linked to decreased MMP‐2/‐9 activity via the MAPK pathway (M. Luo et al. 2017). Zheng et al. developed sino‐wcj‐33 (SW33), a sinomenine derivative, and assessed its anticancer effects on GBM. SW33 significantly reduced U87 and U251 cell migration, invasion, proliferation, and colony formation. It induced mitochondria‐dependent apoptosis and halted the cell cycle at G2/M phase. Furthermore, SW33 reduced tumorigenicity by modulating the PI3K/AKT and AMPK pathways, with no observable adverse effects. It initiated autophagy through the AMPK/mTOR and PI3K/AKT/mTOR pathways, indicating its potential as a safe and effective treatment for GBM (X. Zheng et al. 2021). Galbanic acid significantly inhibited GBM cell proliferation, blocked the cell cycle, and induced apoptosis. It also reduced cell migration, linked to decreased MMP2 and MMP9 expression and activity. The treatment increased PTEN expression while lowering Akt, mTOR, and PI3K gene expression, with no effects on WNT, β‐catenin, or APC genes. Following treatment, p‐Akt protein levels also decreased. Galbanic acid demonstrated effectiveness at lower concentrations than temozolomide. It reduces GBM cell growth and survival by blocking the PI3K/Akt/mTOR pathway and inhibiting MMP activity (Shahcheraghi et al. 2021). Corynoline significantly inhibited the growth of GBM cell lines U87 and LN229, promoting apoptosis. It enhanced proapoptotic proteins Bax, Bak, cleaved caspase‐9, and cleaved caspase‐3, while reducing STAT3 phosphorylation and antiapoptotic protein Bcl2 levels. The STAT3 activator colivelin TFA attenuated these effects by interfering with the STAT3/Bcl2 signaling pathway. Furthermore, corynoline therapy shows promise as a therapeutic option against GBM by effectively targeting the relevant pathway in vivo (Xiong et al. 2026). In GBM cell lines LN‐229 and U87‐MG, resveratrol significantly decreased cell viability, promoted apoptosis, and inhibited proliferation and invasive migration. Its anti‐GBM effects were linked to the JAK/STAT signaling pathway and inflammatory responses. Resveratrol reduced NLRP3 inflammasome over‐activation via the JAK2/STAT3 pathway. The JAK/STAT agonist RO8191 partially reversed this effect. Resveratrol inhibits inflammatory responses in the GBM microenvironment by blocking JAK2/STAT3 activation (Zhang et al. 2024). Eriodictyol inhibits proliferation, migration, and invasion of U87MG and CHG‐5 glioma cells in a dose‐ and time‐dependent manner, inducing apoptosis. It suppresses the PI3K/Akt/NF‐κB signaling pathway, with the PI3K inhibitor LY294002 enhancing its effects on glioma cell death. It also significantly reduces tumor development and induces apoptosis in a mouse xenograft model, effectively blocking glioma cell proliferation and spread (W. Li et al. 2020). TMZ is the only oral first‐line chemotherapy for GBM, but resistance due to MGMT limits its effectiveness. Current small molecule MGMT inhibitors face significant hematological toxicity. This study explores inhibiting MGMT expression by modifying the Wnt/β‐catenin signaling pathway. Thymoquinone effectively reduced MGMT expression in GBM cell lines and showed synergistic effects with TMZ in enhancing cell death and inhibiting migration and proliferation. In a resistant mouse model, thymoquinone improved the efficacy of TMZ while maintaining biosafety. Thymoquinone decreased β‐catenin nuclear translocation and downregulated Cyclin D1 and MGMT expression. It enhances TMZ sensitivity by disrupting the Wnt/β‐catenin pathway (Bai et al. 2025). Cyanidin significantly reduces viability in all glioma stem cell lines, particularly affecting GBM2 cells while showing no impact on 293T cells. It modulates the Wnt signaling pathway across glioma stem cell lines, upregulating WNT1 and MYC in GBM2, GBM7, G166, and G179 but downregulating them in G144 and GliNS2. All lines exhibit decreased β‐catenin levels. Notably, c‐Myc is reduced in GBM2 but remains unchanged in G144 and GliNS2. Cyanidin also lowers Twist1 and Snail1 expression in GBM2, G179, and G144, whereas GliNS2 shows opposite cytoplasmic changes. Overall, cyanidin exerts an antitumor effect on glioma stem cells, likely through the Wnt signaling pathway (Xue et al. 2024).

## 7 Therapeutic Challenges and Nanotechnological Approaches

The clinical viability of natural compounds in treating GBM is influenced by factors such as bioavailability, metabolism, and BBB permeability. This study reveals that several compounds have low oral bioavailability, indicating their inability to enter the systemic circulation after intake. Berberine's rapid first‐pass metabolism and limited absorption lead to low oral bioavailability (C. Liu et al. 2016). Flavonoids quercetin, naringin, and EGCG, due to their high molecular weights and metabolic changes, have poor oral bioavailability (Thilakarathna and Rupasinghe 2013). Brain‐targeting treatments consider both BBB permeability and bioavailability when developing. The BBB facilitates the effective absorption of coumarins and berberine, despite their low oral bioavailability (X. Wang et al. 2005; Y. Yang et al. 2015). Additionally, the BBB allows xanthophylls, lipophilic flavonoids like quercetin and naringin, and arctigenin to pass through the cranium and retina (Widomska et al. 2016; Youdim et al. 2004). The administration of generic yam extract did not significantly pass the flavonoid diosgenin into the rat BBB (Yang et al. 2021). Likewise, paeoniflorin taken orally is unable to cross the mouse BBB (J. Yu et al. 2019). Preclinical GBM research faces challenges due to insufficient modeling of physiological conditions and the BBB, despite natural substance bioavailability and BBB permeability confirmation. Several trials utilized animal models, but some utilized heterotopic xenografts instead of orthotopic ones. Heterotopic xenografts, particularly subcutaneous ones, are not highly effective due to their inability to replicate the BBB. Orthotopic implants accurately model brain tumors, and natural medicines like Astaxanthin, adonixanthin, crocetin, eucalyptol A, tannic acid, rutin, and quercetin show significant anti‐GBM actions in murine orthotopic models (Hua et al. 2021; Colapietro et al. 2020; Tsuji et al. 2020). Recent preclinical research indicates that the selectivity of GBM varies between different drugs and cell lines. Diosmin, a cytotoxic compound, was found to be mildly cytotoxic against human astrocytes at doses up to 150 µM, while tannic acid did not alter the viability of normal rat astrocytes (Bona et al. 2020; SOARES et al. 2019). Human cerebellar astrocytes showed significantly higher rupesin E IC_50_ values than glioma progenitor cells (Qi et al. 2020). Treatment design requires bioavailability, BBB permeability, and GBM selectivity. Curcumin's properties are enhanced by protein complexes, microemulsions, nanosuspensions, and NPs to prevent side effects (Zhai et al. 2020). Dodecamer peptide‐functionalized polydopamine‐coated curcumin‐loaded zein nanoparticles effectively penetrate 3D tumor spheroids and deliver curcumin to GBM cells through the BBB (H. Zhang et al. 2021). Transferrin‐modified osthole liposomes, quercetin polylactide‐co‐glycolide nanoencapsulations, and diosgenin‐olive oil suspensions show superior BBB permeability compared to natural compounds alone (Yang et al. 2021). Photonic NPs within BBB‐permeable structures enable optical monitoring of medication release, specifically following curcumin delivery and distribution (Singh et al. 2016). Furthermore, phototheranostic nanoplatforms have the potential to be utilized for imaging and treating brain tumors (Y. Jia et al. 2018). Chemotherapy traditionally treats gliomas but is limited by low specificity and BBB permeability. Folic acid derivatives and mitochondria‐targeting berberine derivatives were used to construct a liposome, coated with Tween 80, to load paclitaxel (PTX‐Tween 80‐Berberine + Folic Acid‐Liposomes). Folic acid modification targets glioma cells effectively, while berberine is directed to mitochondria, promoting cell death. The liposome's surface is enhanced with Tween 80 to improve BBB penetration. PTX‐Tween 80‐Berberine + Folic Acid‐Liposomes significantly improves chemotherapy efficacy through precise tumor and mitochondrial targeting (C. Yang et al. 2022). Lycopene inhibits the proliferation of GBM8401 and T98G GBM cells by passing the BBB and inducing apoptosis. It enhances p53 expression and inhibits cyclins B and D, leading to cell cycle arrest through ROS‐activated ERK pathways (Ko et al. 2024). A study demonstrates the development of curcumin‐loaded zein NPs functionalized with dodecamer peptide (G23) that effectively cross the BBB to target GBM cells. These NPs enhance cellular absorption of curcumin compared to free curcumin, showing improved penetration into 3D tumor spheroids. They significantly inhibit migration, proliferation, and induce apoptosis in C6 glioma cells. In vivo studies in zebrafish demonstrated circulation of the NPs post‐intravenous injection, indicating potential for targeted drug delivery (H. Zhang et al. 2021). The synergistic effects of paclitaxel and resveratrol‐loaded Soluplus polymeric NPs against glioma cell lines were evaluated. Cytotoxicity tests showed that paclitaxel and resveratrol‐loaded Soluplus polymeric NPs had greater antitumor effects on C6 glioma cells than individual treatments. Pharmacokinetic studies in mice demonstrate that paclitaxel and resveratrol‐loaded Soluplus polymeric NPs have significantly better bioavailability than pure components. This combination significantly enhances anti‐glioma activity in glioma treatment strategy (Hussain et al. 2021). The aggressive GBM poses treatment challenges due to systemic effects and poor drug delivery through the BBB. Innovative nanocarriers, particularly NLCs, improve drug stability and enable targeted delivery. A modified emulsification‐sonication method was used to form NLCs for docetaxel and quercetin, conjugated with transferrin to enhance efficacy. A validated HPLC method demonstrated high accuracy and recovery for the simultaneous detection of docetaxel and quercetin in Wistar rats. Intranasal administration of transferrin (docetaxel and quercetin) NLCs leads to higher brain concentrations of the drugs than intravenous and intranasal suspensions (Qamar et al. 2026).

## 8 Conclusion and Future Perspectives

GBM, despite advancements in surgery, chemotherapy, and radiation therapy, still has a median survival period, making it the most aggressive and challenging primary brain tumor to treat. Additionally, GBM's poor prognosis is primarily due to its invasive growth, cellular heterogeneity, and resistance to conventional therapies. The development of innovative therapeutic strategies heavily relies on addressing the intricate and interconnected pathways involved. Natural compounds are being explored as potential modulators of GBM pathogenesis due to their multitargeted actions and low toxicity profiles. Additionally, natural compounds like curcumin, resveratrol, quercetin, EGCG, and berberine have demonstrated potent anticancer properties by triggering apoptosis, blocking angiogenesis, controlling OS, and preventing invasion and metastasis. These can bypass the BBB, a significant barrier to drug delivery to the CNS. Additionally, the potential synergy with existing radiation and chemotherapeutics highlights their use in combination strategies to address therapeutic resistance. Natural compounds possess both diagnostic and preventative properties, in addition to their therapeutic potential. These alter OS indicators, inflammatory mediators, and molecular biomarkers linked to the advancement of GBM. Advances in molecular diagnostics, like liquid biopsy, genomic profiling, and biomarker‐based imaging, can improve precision medicine strategies by enhancing early detection, disease monitoring, and therapy response forecasting. Despite promising preclinical results, there are numerous obstacles to the clinical translation of natural drugs. Therapeutic effectiveness is often compromised by their limited stability, fast metabolism, and poor bioavailability. Nanotechnology‐based delivery systems, structural modifications, and formulations are being developed to improve tumor specificity and pharmacokinetics, addressing existing issues. However, to ensure their safety and effectiveness in human populations, extensive, precisely monitored clinical trials are required for thorough validation. The inclusion of natural compounds in mainstream medicine faces challenges such as overcoming variations in purity, standardization, and regulation. Further mechanistic research is essential to accurately understand the interactions between natural substances and GBM‐specific signaling networks, as well as the tumor microenvironment, including immune cells and stromal factors. Prioritize combining natural compounds with immunotherapies, targeted medicines, or traditional chemotherapeutics to maximize synergistic effects. To enhance brain penetration and minimize systemic toxicity, it is recommended to optimize nanocarrier systems like liposomes, polymeric NPs, and exosome‐based delivery vehicles. Natural compounds targeting signaling pathways present a promising new approach for GBM treatment. Bioactive compounds provide benefits over conventional single‐target drugs by affecting multiple oncogenic pathways and demonstrating neuroprotective properties.

## Author Contributions

**Mehrukh Zehravi**: conceptualization, data curation, formal analysis, investigation, methodology, resources, software, supervision, visualization, roles/writing – original draft, writing – review and editing. **Md Abul Hassan**: data curation, investigation, methodology, resources, software, roles/writing – original draft, writing – review and editing, supervision. **Md. Al Amin**: data curation, investigation, methodology, resources, software, roles/writing – original draft, writing – review and editing. **Sherouk Hussein Sweilam**: data curation, investigation, methodology, resources, software, roles/writing – original draft, writing – review and editing. **Jeetendra Kumar Gupta**: investigation, validation, visualization, roles/writing – review and editing. **Ramenani Hari Babu**: formal analysis, investigation, validation, visualization, roles/writing – review and editing. **Pericharla Venkata Narasimha Raju**: formal analysis, investigation, validation, visualization, roles/writing – review and editing. **Krishna Vamsi Kandimalla**: formal analysis, investigation, validation, visualization, roles/writing – review and editing. **Phanindra Erukulla**: formal analysis, investigation, validation, visualization, roles/writing – review and editing. **Patibandla Jahnavi**: formal analysis, investigation, validation, visualization, roles/writing – review and editing. **Rajeshwar Vodeti**: formal analysis, investigation, validation, visualization, roles/writing – review and editing. **Abdul Ajeed Mohathasim Billah**: formal analysis, investigation, validation, visualization, roles/writing – review and editing. **Safia Obaidur Rab**: formal analysis, investigation, validation, visualization, roles/writing – review and editing. **Sharuk L. Khan**: conceptualization, formal analysis, funding acquisition, investigation, methodology, project administration, supervision, validation, visualization, writing – review and editing. All authors have read and agreed to the published version of the manuscript.

## Ethics Statement

The authors have nothing to report.

## Consent

The authors have nothing to report.

## Conflicts of Interest

The authors declare no conflicts of interest.

## Data Availability

All data supporting the findings of this study are available in the paper.
